# Investigating the K^+^ sensitivity of cellular metabolism by extracellular flux analysis

**DOI:** 10.1016/j.xpro.2021.100876

**Published:** 2021-11-05

**Authors:** Sandra Burgstaller, Helmut Bischof, Robert Lukowski, Wolfgang F. Graier, Roland Malli

**Affiliations:** 1 Gottfried Schatz Research Center, Molecular Biology and Biochemistry, Medical University of Graz, Neue Stiftingtalstraße 6/6, 8010 Graz, Austria; 2 Department of Pharmacology, Toxicology and Clinical Pharmacy, Institute of Pharmacy, University of Tuebingen, Auf der Morgenstelle 8, 72076 Tuebingen, Germany; 3 Natural and Medical Sciences Institute, University of Tuebingen, 72770 Reutlingen, Germany; 4 BioTechMed-Graz, Mozartgasse 12/II, 8010 Graz, Austria

**Keywords:** Cell Biology, Cell-based Assays, Cancer, High Throughput Screening, Metabolism, Molecular Biology

## Abstract

We have recently demonstrated that the activity of hexokinase 2 is dependent on the intracellular potassium ion (K^+^) concentration ([K^+^]). To analyze the K^+^ dependency of the cell metabolism in cell populations, we used an extracellular flux analyzer to assess oxygen consumption and acidification rates as well-established measures of oxidative- and glycolytic metabolic activities. This protocol describes in detail how a potential K^+^ sensitivity of the cell metabolism can be elucidated by extracellular flux analysis.

For complete details on the use and execution of this protocol, please refer to Bischof et al. (2021).

## Highlights

• The K^+^ sensitivity of cell metabolism is investigated by extracellular flux analysis
• Intracellular K^+^ depletion decreases the ratio of glycolytic/ oxidative activity
• Glycolytic activity is maintained by high extracellular K^+^ concentrations

## Graphical abstract

**Figure:**
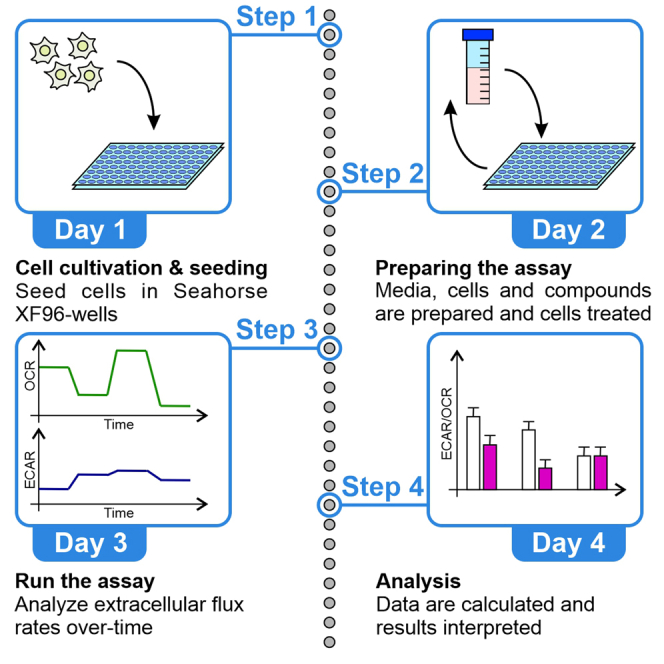

## Before you begin

The workflow described in this protocol takes 2 days with requirements of specific equipment (see materials and equipment). Before starting the experiments, specified media must be prepared, and a sufficient number of cells, which should be maintained on one 10 cm cell culture dish should be available for cell seeding on day 1. A confluency of 80%–90% can be considered sufficient for conducting the experiments described. The protocol described herein focuses on the analysis of HEK293, HeLa, and INS-1 832/13 cells. However, this protocol has also been tested using established breast cancer cell lines such as MCF-7 and MDA-MB-453 cells.

Having identified recently that hexokinase-II (HKII) represents an enzyme depending on K^+^ (Bischof et al., 2021), this protocol aims to demonstrate how to unveil potential K^+^ sensitive structures/ enzymes in cell metabolic activity and allows to determine the contribution of HKII to the metabolic homeostasis in diverse (primary) cell lines. A strong affection of extracellular acidification rates (ECAR), i.e., glycolytic activity, by intracellular K^+^ depletion indicates a significant contribution of HKII for the maintenance of the glycolytic activity in these cells. An additional affection of oxygen consumption rates (OCR), i.e., oxidative phosphorylation, by intracellular K^+^ depletion points to a significant contribution of glycolytic substrates to the oxidative phosphorylation activity.

### Culture cell lines

**Timing: Day 0**

The cells described herein can be handled without the need for special (pre-) treatment. For the cultivation of HEK293 and HeLa cells, DMEM with 10% fetal calf serum (FCS) was supplemented with 1**×** Penicillin-Streptomycin and 1**×** sodium pyruvate, while INS-1 832/13 cells were cultivated in Gibco RPMI 1640 medium containing 10% FCS, 10 mM HEPES, 0.05 mM 2-mercaptoethanol, 1**×** sodium pyruvate, 1**×** Penicillin-Streptomycin. For the cultivation of all cell lines, a humidified incubator adjusted to 37°C and 5% CO_2_ was used. Cell splitting procedures were adjusted to the cell line specific growth properties and initial seeding density and were carried out when the confluency reached 80%–90%. For cell detachment, Trypsin-EDTA at a final concentration of 0.05% in PBS was used.

**CRITICAL:** Adjust the splitting ratios to the cell lines. For HEK293, HeLa and INS-1 832/13 cells, splitting ratios of 1:6 are recommended. Other cell lines might need to be split into lower or higher ratios to ensure growth and viability. Do not conduct metabolic experiments with cells at very high passages, e.g. above p50. Finally, the aseptic culture conditions should be as consistent as possible with no disturbances in the temperature or CO_2_ levels.

### Preparation of the media used for extracellular flux analysis

**Timing: Day 0**

To investigate the K^+^ sensitivity of cellular metabolism by extracellular flux analysis as well as its putative reversibility, two different media need to be prepared. The first medium contains 0 mM K^+^ and is referred to as “Seahorse Medium without K^+^ ” (SM-0K^+^ ), the second medium contains 140.0 mM K^+^ and is referred to as “Seahorse medium-high potassium” (SM-140K^+^ ). The formulation of these media can be found in the “materials and equipment” section below.

## Key resources table

**Table:** 

REAGENT or RESOURCE	SOURCE	IDENTIFIER
**Chemicals, peptides and recombinant proteins**		

2-Deoxy-D-glucose	Tocris	n/a
2-Mercaptoethanol	Thermo Fisher Scientific	n/a
50**×** MEM Amino Acids Solution	Thermo Fisher Scientific	n/a
100**×** MEM Vitamin Solution	Thermo Fisher Scientific	n/a
100**×** Sodium Pyruvate Solution	Thermo Fisher Scientific	n/a
Antimycin-A	Abcam	n/a
CaCl_2_2H_2_O	Carl Roth	n/a
Carbonyl cyanide 4-(trifluoromethoxy)phenylhydrazone (FCCP)	Santa Cruz Biotechnology	n/a
D-Glucose H_2_O	Carl Roth	n/a
DMEM, high glucose, HEPES	Thermo Fisher Scientific	n/a
FeCl_2_	Sigma-Aldrich	n/a
Fetal Calf Serum	Thermo Fisher Scientific	n/a
Gibco RPMI 1640 Medium	Thermo Fisher Scientific	n/a
Gramicidin	Sigma-Aldrich	n/a
HEPES	Carl Roth	n/a
KCl	Carl Roth	n/a
KHCO_3_	Carl Roth	n/a
KH_2_PO_4_	Carl Roth	n/a
KOH	Carl Roth	n/a
L-Glutamine (200 mM)	Thermo Fisher Scientific	n/a
MgSO_4_	Carl Roth	n/a
NaCl	Carl Roth	n/a
NaHCO_3_	Carl Roth	n/a
NaH_2_PO_4_	Carl Roth	n/a
NaOH	Carl Roth	n/a
Oligomycin-A	Tocris	n/a
Penicillin-Streptomycin (100**×**)	Thermo Fisher Scientific	n/a
Phenol red solution	Sigma-Aldrich	n/a
Phosphate buffered saline	Thermo Fisher Scientific	n/a
Rotenone	Tocris	n/a
Trypsin-EDTA (0.5%)	Thermo Fisher Scientific	n/a

**Critical commercial assays**		

Pierce BCA Protein Assay Kit	Thermo Fisher Scientific	n/a
Seahorse XFe96 Flux Pak	Agilent Technologies	n/a

**Experimental models: cell lines**		

HEK293	ATCC	n/a
HeLa S3	ATCC	n/a
INS-1 832/13	Gift from C.B. Newgard (Department of Pharmacology and Cancer Biology, Duke University School of Medicine, USA)	n/a

**Software and algorithms**		

Excel Office 365	Microsoft	n/a
Prism 8	GraphPad	n/a
Seahorse Wave Desktop Software	Agilent Technologies	n/a

**Other**		

15 mL Centrifuge Tubes	Corning	n/a
37°C Incubator without CO_2_	Binder	Cat. No. B28
37°C Incubator, 5% CO_2_, humidified	Thermo Fisher Scientific	Cat. No. 51026331
37°C Water bath	Phoenix WB-22	n/a
Bürker-Türk cell counting chamber	Merck	Cat. No. MDH-2N1-50PK
Centrifuge	Eppendorf	Cat. No. 5702000010
Seahorse XF96 Analyzer	Agilent Technologies	n/a
Seahorse XF96 Cell Culture Microplates	Agilent Technologies	Cat. No. 101085-004
Seahorse XF96 FluxPak	Agilent Technologies	Cat No. 102416-100
Sterile workbench	Thermo Fisher Scientific	HS12

## Materials and equipment

To minimize alterations in the buffer formulations, we recommend the use of stock solutions.

**Table:** Formulation of cell culture medium used for culturing HEK293 and HeLa cells. Before adding supplemental compounds, remove 60 mL of the respective DMEM, medium from the bottle.

Reagent	Final concentration	Amount
100**×** Penicillin-Streptomycin	1**×**	5 mL
100**×** sodium pyruvate	1**×**	5 mL
DMEM, high Glucose, HEPES	n/a	440 mL
FCS	10%	50 mL
**Total**	**n/a**	**500 mL**

**Table:** Formulation of cell culture medium used for culturing INS-1 832/13 cells. Before adding supplemental compounds, remove 65 mL of the Gibco RPMI 1640 medium from the bottle.

Reagent	Final concentration	Amount
2-mercaptoethanol	0.05 mM	2 μL
100**×** Penicillin-Streptomycin	1**×**	5 mL
100**×** sodium pyruvate	1**×**	5 mL
FCS	10%	50 mL
Gibco RPMI 1640 medium	n/a	435 mL
HEPES	10 mM	5 mL of 1.0 M
**Total**	**n/a**	**500 mL**

**Table:** Formulation of seahorse medium – without potassium (SM-0K^+^)

Reagent	Final concentration	Amount
CaCl_2_ 2H_2_O	1.8 mM	9 mL of 0.1 M
FeCl_2_	0.000248 mM	12.8 μL of 0.01 M
MgSO_4_	0.814 mM	4.07 mL of 0.1 M
NaCl	115.36 mM	57.68 mL of 1.0 M
NaHCO_3_	44.0 mM	22 mL of 1.0 M
NaH_2_PO_4_	0.908 mM	4.54 mL of 0.1 M
Phenol Red	0.0005%	500 μL of Stock
MEM Amino Acids	1**×**	10 mL of 50**×**
MEM Vitamins	1**×**	5 mL of 100**×**
ddH_2_O	n/a	387.45 mL
**Total**	**n/a**	**500 mL**

After sterile filtration of the freshly prepared SM-0K^+^ medium, it can be stored for up to 2 months in the dark at 4°C.

**Table:** Formulation of the seahorse medium – high potassium (SM-140K^+^)

Reagent	Final concentration	Amount
CaCl_2_ 2H_2_O	1.8 mM	9 mL of 0.1 M
FeCl_2_	0.000248 mM	12.8 μL of 0.01 M
KCl	115.36 mM	57.68 mL of 1.0 M
KHCO_3_	44.0 mM	22 mL of 1.0 M
KH_2_PO_4_	0.908 mM	4.54 mL of 0.1 M
MgSO_4_	0.814 mM	4.07 mL of 0.1 M
Phenol Red	0.0005%	500 μL of Stock
MEM Amino Acids	1**×**	10 mL of 50**×**
MEM Vitamins	1**×**	5 mL of 100**×**
ddH_2_O	n/a	387.45 mL
**Total**	**n/a**	**500 mL**

After sterile filtration of the freshly prepared SM-140K^+^ medium, it can be stored for up to 2 months in the dark at 4°C.

**Table:** Equipment.

Component	Note
37°C incubator without CO_2_	For equilibration of seahorse cell culture plate before the experiment
37°C incubator, 5% CO_2_, humidified	For cell culture cultivation
37°C water-bath	For pre-warming of media
Bürker-Türk cell counting chamber	For counting cells
Centrifuge	For pelleting cells
Seahorse XF96 Analyzer	For measurements of the extracellular acidification rate and the oxygen consumption rate. Other Seahorse devices may be used, such as the Seahorse XF24, Seahorse XFe24 or Seahorse XFe96.
Seahorse XF96 Cell Culture Microplates	For cultivation of cells of interest
Seahorse XF96 Sensor Cartridges	For Seahorse experiments
Sterile workbench	For cell culture, seeding, and handling

## Step-by-step method details

### Designing the experiment

**Timing: Day 1, 0.5 h**

Before plating the cells the experiment needs to be designed using the Seahorse Wave Desktop Software.

1. Start the Seahorse Agilent Wave Desktop Software
  a. The software can be downloaded from https://www.agilent.com/en/products/cell-analysis/software-download-for-wave-desktop.
  b. Install the software and run the application.
2. Design the experiment and define groups.
  a. Open the “blank” file under the templates section.
  b. Define the conditions of the experiment in the “definitions” section. Start with defining the injection strategies, pretreatments, assay media, and cell type.
    i. Injection strategies might be neglected if only basal (no injection) extracellular acidification rates (ECAR) or basal oxygen consumption rates (OCR) are of interest. Depending on the experimental setups, injection strategies might be added.
    ii. Define pretreatments, which will additionally comprise the treatment with DMSO as control and Gramicidin (dissolved in DMSO) as the actual experimental condition. Further pretreatments might be added, if necessary.
    iii. Define the assay media used, which will comprise SM-0K^+^ and SM-140K^+^ medium. Further assay media might be added, if necessary.
      Cell treatment with the pore forming peptide gramicidin allows to adjust the intracellular K^+^ concentration ([K^+^]) to the [K^+^] present in the extracellular space/ medium, i.e., 0 mM K^+^ (SM-0K^+^) and 140.0 mM K^+^ (SM-140K^+^).
    iv. Define the cell type used in the experiments, for example, HEK293, HeLa, and INS-1 832/13.
  c. Define your treatments or manipulations in the “groups” section. Definitions need to be precise and consistent.
    i. In the beginning, only a “background” group will be defined, which can neither be altered nor removed, as it is indispensable.
    ii. For each condition (12 in this case, comprising HEK293, HeLa, and INS-1 832/13, each treated with SM-0K^+^ DMSO, SM-0K^+^ GRAM, SM-140K^+^ DMSO, and SM-140K^+^ GRAM, respectively) add a new group and choose the respective injection strategy, pretreatments, assay media, and cell type. Accurately defining the groups will lead to sorted results after the assay is finished and the results have been exported. The names of the groups can be changed by a right-click on the group header. The “Generate Groups” function in the Seahorse Wave Desktop software can automatically assign the groups, if the pretreatments, assay media and cell types have been correctly defined.
  d. Assign the defined groups to the wells by clicking the group on the left side and clicking the wells comprising the respective groups on the right side. The “distribute groups” button might be used and will fairly spread the groups on the plate. We recommend including at least four blanks on the plate. The software automatically suggests using the 4 edge wells as a background.
  e. Define the protocol that is used for analyzing the cells. If only basal ECAR/OCR ratios are of interest, the measurement of a baseline without injection is sufficient. The protocol might, however, be altered depending on the desired read-out, and injections at different time points might be added. We recommend performing at least 4 measurement cycles.
  f. Once the protocol is defined, it can be saved and re-opened when the measurement is started.

**CRITICAL:** Defining groups accurately and carefully will help you identify the experimental conditions long after the experiment was performed and will directly show you the results online on the device.

### Cell counting and seeding

**Timing: Day 1, 2 h**

**Figure 1:**
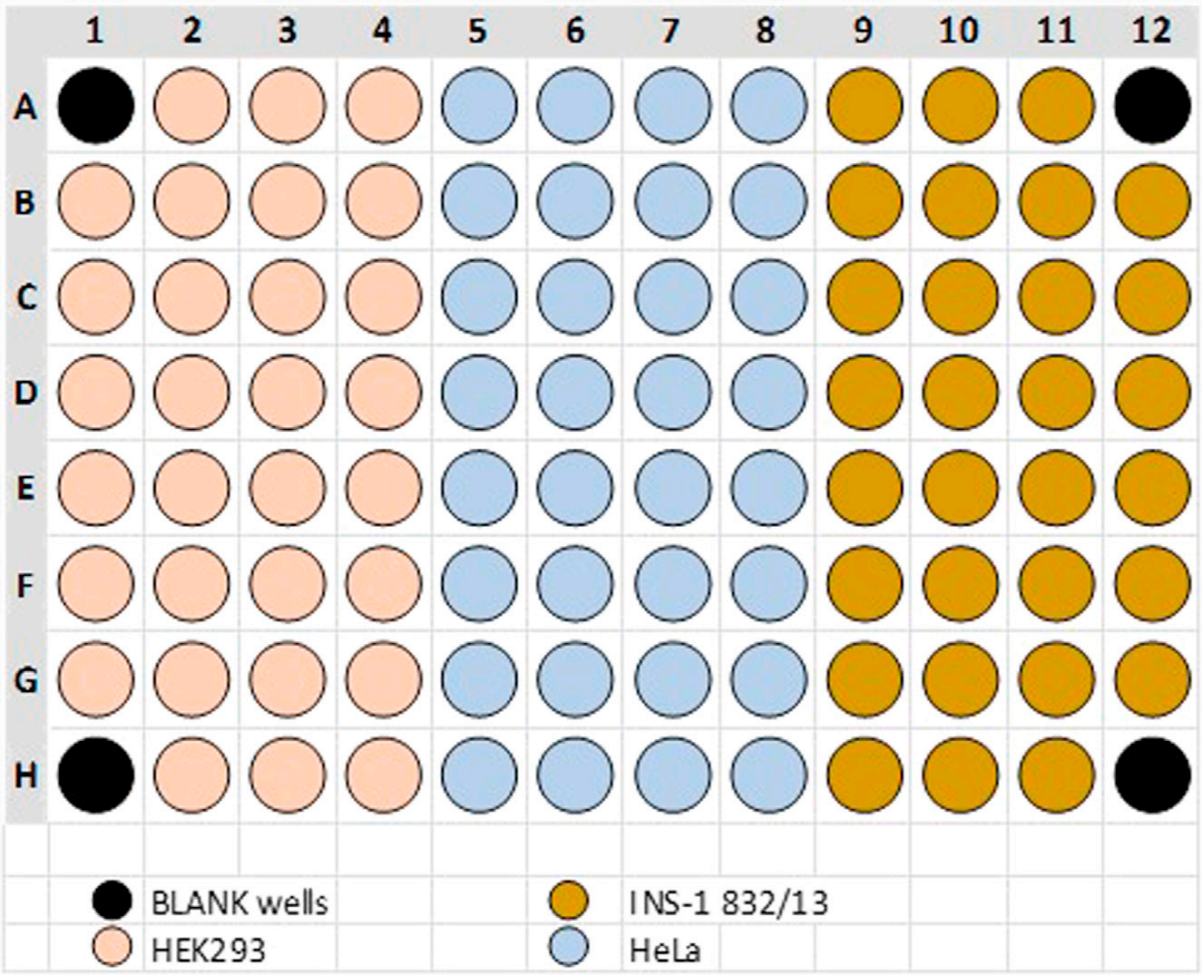
Typical layout recommended for cell seeding

The cell number which needs to be seeded into the wells of a Seahorse XF96 Cell Culture Microplate depends on the size of the adherent cells. Typically, for HEK293, HeLa, and INS-1 832/13 cells, 70.000, 50.000, and 100.000 cells, respectively, yield a confluency of ∼90% on the next day, which can be checked by a conventional bright-field microscope. Such confluency is a good starting point for performing the experiments. Typical cell numbers seeded per well of a 24- or 96-well plate can be found in Table 1. A typical layout for cell seeding is demonstrated in Figure 1.

3. Remove the cells, cultivated in a 10 cm cell culture dish (confluency of ∼ 90%), from the incubator and trypsinize cells under sterile conditions.
  a. After removing the cells from the incubator, aspirate the medium and wish twice with 6–10 mL pre-warmed PBS.
  b. Aspirate PBS and add 2 mL of pre-warmed trypsin-EDTA at a concentration of 0.05% in PBS to the cells and put the cell back into the incubator.
  c. Check for cell detachment after 3 min at 37°C, 5% CO_2_ using a cell culture microscope. If cells appear round shaped, they detached from the surface of the culture dish, proceed to 3d. If cells are still attached, prolong the treatment in trypsin-EDTA/PBS but check every minute for cell detachment.
  d. After detachment, stop the enzymatic reaction immediately by adding at least the five- to the six-fold amount of the respective cell culture medium with 10% FCS (FCS will stop the reaction).
  e. Transfer the cell suspension to a 15 mL conical tube and centrifuge for 5 min at 200 rcf.
  f. Aspirate the supernatant without disturbing the cell pellet. Resuspend the cells in 10 mL of cell culture full medium (DMEM + 10% FCS / RPMI 1640 + 10% FCS).
4. To seed similar amounts of cells, the cell number must be determined. This can be done by either using a Bürker-Türk counting chamber or any other method of choice (e.g., CASY cell counter).
  a. When using a Bürker-Türk counting adjust the glass plate to the chamber and fill each of the two chambers (on top and bottom) with 15 μL of the cell suspension. Take care to resuspend the cells thoroughly before pipetting them into the chamber.
  b. While different counting methods exist, we mainly counted the four large squares on the edges, with each square consisting of 16 smaller squares. Count all cells visible in the four large squares and divide this number by 4. Take the average number counted from the top and bottom chamber.
  c. This number has to be multiplied by 10,000, which refers to the number of cells per milliliter cell suspension.
5. Seed HEK293, HeLa, and INS-1 832/13 cells into the wells of a Seahorse XF96 Cell Culture Microplate as in the layout as previously defined in the group definition (step 2c and Figure 1) and cultivate over-night.
  a. Prepare appropriate cell suspension dilutions.
    i. For HEK293 cells, 70.000 cells should be seeded per well. If cells are cultivated in a final volume of 125 μL, adjust the number of cells to 560.000 / milliliter by diluting the cells with full medium (DMEM + 10% FCS).
    ii. For HeLa cells, 50.000 cells should be seeded per well. If cells are cultivated in a final volume of 125 μL, adjust the number of cells to 400.000 / milliliter by diluting the cells with full medium (DMEM + 10% FCS).
    iii. For INS-1 832/13 cells, 100.000 cells should be seeded per well. If cells are cultivated in a final volume of 125 μL, adjust the number of cells to 800.000 / milliliter by diluting the cells with full medium (RPMI 1640 + 10% FCS).
  b. Transfer 125 μL of the diluted cell suspension into each well of the Seahorse XF96 Cell Culture Microplate. Leave the 4 corner wells as a blank and do not seed cells into the edges!
  c. Add 125 μL of full medium (DMEM + 10% FCS) into the 4 wells on the edges, which will serve as a blank during the measurement.
  d. Once cells are seeded, put the Seahorse XF96 Cell Culture Microplate in a humidified incubator at 37°C with 5% CO_2_ and cultivate the cells 14–20 h to allow cell attachment.

**CRITICAL:** Keep the wells on the edges of the Seahorse XF96 Cell Culture Microplate free from cells and add the same amount of complemented medium (DMEM + 10% FCS / RPMI 1640 + 10% FCS) instead, as these wells will serve as a blank during the measurement. The number of cells that need to be seeded to achieve a density of ∼ 90% on the next day highly depends on the cell type. For smaller cells as well as slowly growing cells, a higher cell number might be seeded.

**Table 1:** Typical cell numbers used for seeding in Seahorse XFe96 or XFe24 cell culture plates

Cell type / Cell line	Seeded cell number / well	
	Seahorse XFe96 cell culture plates	Seahorse XFe24 cell culture plates
HEK293	70.000	100.000
HeLa	50.000	70.000
INS-1 832/13	100.000	140.000
MCF-7	50.000	70.000
MDA-MB-453	80.000	110.000

### Hydration of the sensor cartridge

**Timing: Day 1, 10 min**

The sensor cartridge of the Seahorse XF96 Analyzer needs to be hydrated one day (6–48h) before the experiment.

6. Hydrate the Seahorse XF96 Sensor Cartridge according to the manufacturer's instructions.
  a. Remove the Seahorse XF96 Sensor Cartridge (green plate) from the 96-well plate (clear plate) with taking care of the sensor modules. Put the plate on the back so that the sensor modules look upwards and do not touch the ground.
  b. Add 100 μL of calibrant solution delivered with the Seahorse XF96 Sensor Cartridges into every well of the clear 96-well plate.
  c. Re-insert the Seahorse XF96 Sensor Cartridge (green plate) into the 96-well plate. Every sensor should be covered with a calibrant solution.
  d. Place the hydrated Seahorse XF96 Sensor Cartridge in a 37°C incubator WITHOUT CO_2_ and incubate 14–20 h.
7. Pre-warm the Seahorse XF96 Analyzer
  a. Switch on the Seahorse XF96 Analyzer and start the controlling software one day before the experiment to allow the device to pre-warm.
  b. Check the display of the device to ensure it is pre-heating.

Additional information about sensor hydration can be found at the page of the manufacturer – see https://www.agilent.com/cs/library/usermanuals/public/XFe96_DAY_BEFORE_CARTRIDGE_HYDRATION.pdf

**CRITICAL:** Avoid any damage to the sensors on the Seahorse XF96 Sensor Cartridge and remember to switch on the Seahorse device.

### Complementation and preparation of assay media

**Timing: Day 2, 30 min**

Before conducting the assay, the respective Seahorse assay media need to be complemented by adding the desired amount of D-glucose, L-glutamine, and pyruvate. Additionally, the pH needs to be adjusted to 7.40 at 37°C to get consistent results.

8. Transfer 48.725 mL of both Seahorse assay media, SM-0K^+^ and SM-140K^+^, into a sterile 50 mL falcon under sterile conditions and place in a 37°C water bath.
9. Once media are warmed, the desired amounts of D-glucose, L-glutamine, and pyruvate can be added.
  a. As a starting point, we recommend using 5.5 mM D-glucose, 2 mM L-glutamine, and 1 mM sodium pyruvate.
    i. We also recommend using stock solutions to avoid dilution errors and to improve the accuracy of pipetting. Therefore, 275 μL of 1.0 M D-glucose, 500 μL of 100**×** L-glutamine, and 500 μL of 100**×** pyruvate should be added to 50 mL medium.
10. Adjust the pH of the complemented media to 7.40.
  a. The media can be placed in a beaker containing a stir bar and a thermometer on a magnetic stirrer equipped with a heating unit. Keep the temperature of the medium at 37°C during pH adjustment.
  b. When 37°C is reached, adjust the pH of the medium to 7.40 using 0.1 N NaOH for SM-0K^+^ or KOH for SM-140K^+^ medium. Add NaOH or KOH dropwise as the media are weakly buffered and be careful not to overshoot the desired pH.
11. Prepare the media containing gramicidin or equivalent amounts of DMSO as a control.
  a. Transfer 2**×**10 mL of both media (SM-0K^+^ and SM-140K^+^) into 15 mL tubes and add gramicidin at a final concentration of 5 μM to the SM-0K^+^ and SM-140K^+^ medium.
    i. We recommend preparing a 15 mM gramicidin stock solution in DMSO. Add 3.33 μL of this solution to each of the two vials containing the SM-0K^+^ and SM-140K^+^ medium, yielding a final gramicidin concentration of 5 μM. Using 5 μM of the pore-forming peptide gramicidin allows to permeabilize cells for K^+^ and to adjust the intracellular [K^+^] to the [K^+^] present in the extracellular space/ medium.
  b. Transfer 2**×**10 mL of both media (SM-0K^+^ and SM-140K^+^) into 15 mL tubes and add equivalent amounts of DMSO to the SM-0K^+^ and SM-140K^+^ medium.
    i. If a 15 mM gramicidin stock was used, add 3.33 μL of DMSO to each of the two vials containing the SM-0K^+^ and SM-140K^+^ medium, yielding a final DMSO concentration of 0.033%.
12. Keep all media at a temperature of 37°C using a water bath until usage.

**CRITICAL:** Adjust the pH to 7.40 for both media, SM-0K^+^ and SM-140K^+^ , carefully and slowly as the pH will alter very fast and the pH must not be re-arranged. Take care to adjust the pH at 37°C, as the assay is performed at this temperature. Adjusting pH at other temperatures will cause inaccurate measurements.

***Optional:*** Keep the medium free from D-glucose and inject glucose via the ports of the Seahorse XF96 Sensor Cartridge for being able to investigate the levels of non-glycolytic acidification. For more details see https://www.agilent.com/cs/library/usermanuals/public/XF_Glycolysis_Stress_Test_Kit_User_Guide.pdf.

### Loading sensor cartridge with modulators

**Timing: Day 2, 30 min**

Depending on the information you want to receive from the experiment, you may treat cells with modulators of glycolysis or oxidative phosphorylation. To receive information about the effects of K^+^ depletion on the oxidative phosphorylation activity, oligomycin-A, an ATP-synthase inhibitor, FCCP, a mitochondrial uncoupler, antimycin-A, a mitochondrial complex III inhibitor, and rotenone, a mitochondrial complex I inhibitor may be injected. For receiving more information about the glycolytic activity, D-glucose, oligomycin-A, and 2-deoxy-D-glucose (2-DG) may be injected. For more details about this procedure see https://www.agilent.com/cs/library/usermanuals/public/XF_Cell_Mito_Stress_Test_Kit_User_Guide.pdf and https://www.agilent.com/cs/library/usermanuals/public/XF_Glycolysis_Stress_Test_Kit_User_Guide.pdf.

### Loading sensor cartridge into the seahorse device

**Timing: Day 2, 10 min**

The Seahorse XF96 Sensor Cartridge and the Seahorse XF96 Cell Culture plate are put separately into the Seahorse XF96 Analyzer, with Seahorse XF96 Sensor Cartridge loaded first.

13. Remove the hydrated Seahorse XF96 Sensor Cartridge from the 37°C incubators and put it into the Seahorse Analyzer.
  a. Run the experiment that you have already designed with the Seahorse Wave Desktop Software on the Seahorse XF96 Analyzer. Once you click “run experiment”, the device will ask you to insert the hydrated sensor cartridge on top of the utility plate.
  b. Remove the lid from the plate and put the hydrated Seahorse XF96 Sensor Cartridge into the device, with well A1 being the rear position on the left-back.
  c. Confirm the insertion of the Seahorse XF96 Sensor Cartridge. The device will close and start equilibrating the plate automatically.

**CRITICAL:** Remove the lid from the Seahorse XF96 Sensor Cartridge before putting it into the device. Not removing the lid will not give you results and might damage the device.

### Preparing cells for the assay

**Timing: Day 2, 1 h**

**Figure 2:**
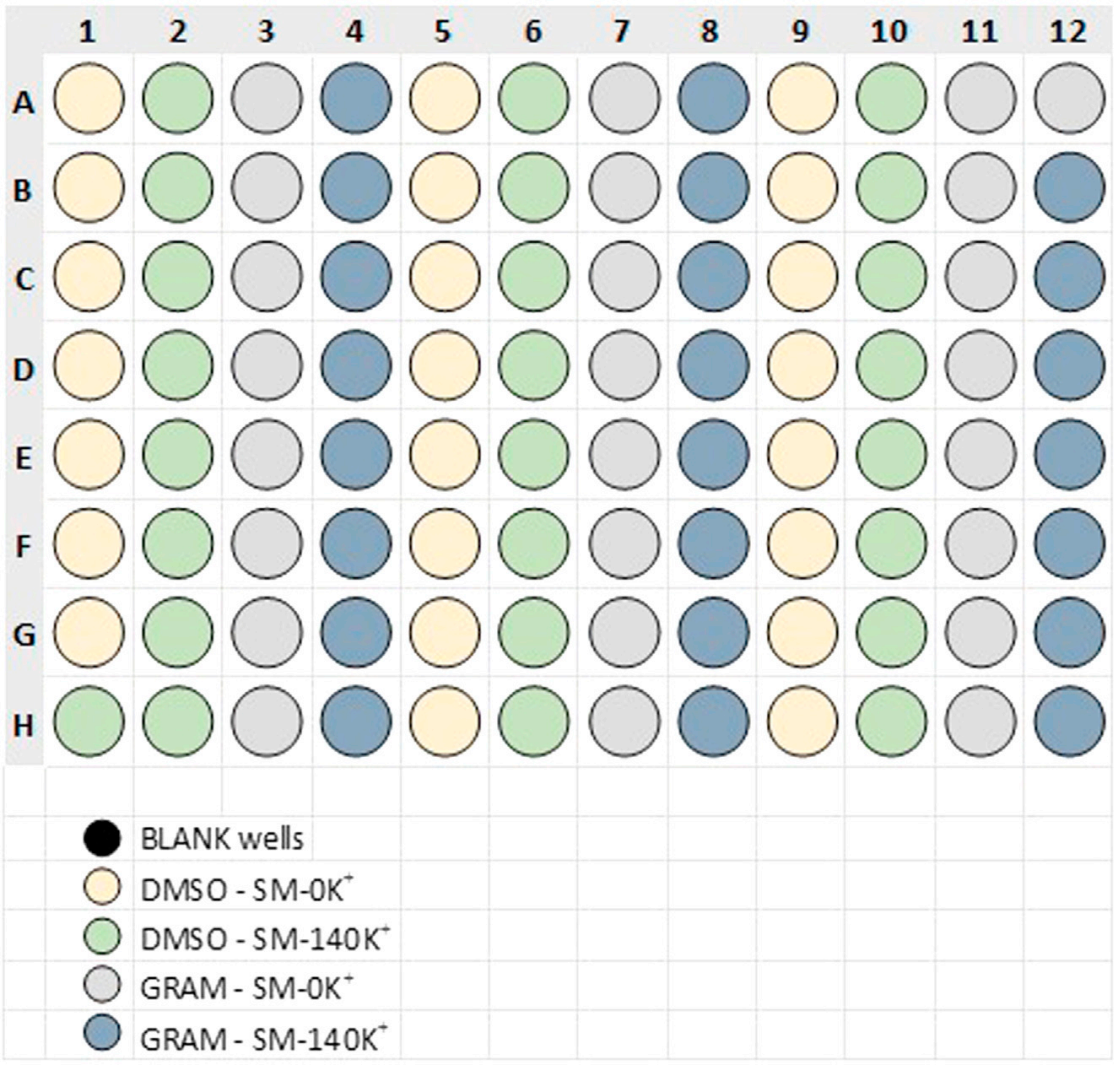
Typical layout recommended for cell treatment with the different media

Before starting the assay, cells need to be washed with the freshly prepared assay media and should be equilibrated. In case of seeding the same cell type in all 96-wells of the Seahorse XF96 cell culture plate, medium of the half of the plate should be exchanged for SM-0K^+^ medium to remove residual K^+^ from the culture medium and the medium of the other half of the plate should be exchanged for SM-140K^+^ medium to adjust the [K^+^] to the desired high concentration. Figure 2 shows a typical compound-layout if cells were seeded as demonstrated in Figure 1.

14. Prepare the cells for the extracellular flux analysis assay.
  a. Remove the cells from the incubator (humidified, 37°C, 5% CO_2_) and check the wells for cell confluency. Ideally, cells show a confluency of ∼90%. Troubleshooting 1.
  b. If cells show a confluency of ∼90%, exchange medium twice with SM-0K^+^ or SM-140K^+^ buffer. One half of the plate should be treated with SM-0K^+^ medium, the other half with SM-140K^+^ medium.
    i. First, remove the culture medium from the cells, but leave 25 μL remaining in the wells and subsequently add 125 μL of the respective assay medium (SM-0K^+^, SM-140K^+^, both without DMSO or gramicidin).
    ii. Second, remove the150 μL from the wells and add 150 μL of the respective assay medium (SM-0K^+^, SM-140K^+^, both without DMSO or gramicidin).
  c. Treat the cells with DMSO and gramicidin to modulate their intracellular [K^+^].
    i. Remove 150 μL of the media and add SM-0K^+^ + DMSO medium or SM-0K^+^ + gramicidin medium to half of the wells washed with SM-0K^+^ medium before.
    ii. Remove 150 μL of the media from the cells and add SM-140K^+^ + DMSO medium or SM-140K^+^ + gramicidin medium to half of the wells washed with SM-140K^+^ medium before.
    iii. Check the wells for the presence and attachment of the cells within the wells using a microscope. As many washing steps were performed, the cells might detach.
  d. Place the cells in a 37°C incubator without CO_2_ for 20 min to allow cell permeabilization by gramicidin, which leads to an adjustment of the intracellular [K^+^] to the extracellular [K^+^].
  e. After 20 min of incubation, remove the plate from the incubator and replace the SM-0K^+^ and SM-140K^+^ medium containing DMSO / gramicidin with the respective assay medium without DMSO and gramicidin.
    i. Carefully remove all 150 μL from the plate without disturbing or scraping the cells and replace with 150 μL of SM-0K^+^ or SM-140K^+^ free from DMSO, respectively.
  f. Incubate the cells at 37°C without CO_2_ for 10 min.

### Running the assay

**Timing: Day 2, variable, depending on the protocol**

While preparing the cells for the assay and after loading the Seahorse XF96 Sensor Cartridge, the device will ask you for the Seahorse XF96 Cell Culture plate. Load the Seahorse XF96 Cell Culture plate when all incubation steps have been finished.

15. Run the assay.
  a. Once the device asks you for the Seahorse XF96 Cell Culture plate, insert the plate after incubation times are completed and confirm the insertion of the plate to start the experiment.
  b. During the experiment, the device will show you the measured values. Troubleshooting 2, 3, 4, and 5.

**CRITICAL:** The Incubation times of cells should not be shortened. Put the plate into the device immediately after the respective incubation times. Avoid spill over from the different wells during repetitive handling of the plates.

### Finishing the assay

**Timing: Day 2, 10 min**

Finish the assay by following the steps on the screen of the Seahorse XF96 Analyzer.

16. Once you confirm to finish the assay, the Seahorse XF96 Sensor Cartridge will move out of the device on top of the Seahorse XF96 Cell Culture plate. Both plates can be discarded.

***Optional:*** Do not discard the Seahorse XF96 Cell Culture plate if you want to normalize data for protein concentration (see step 17b) or other parameters of choice (i.e. DNA content, cell number or protein concentration). Data normalization is necessary if raw OCR and ECAR values are of interest. If no cell pre-treatment was performed, one can assume that cell numbers from well to well will not vary and data can be normalized for the seeded cell number. Alternatively, in cases where cell detachment after washing was observed, Seahorse data can be normalized for the protein concentration per well to correct for reduced cell numbers.

### Normalize OCR and ECAR values

**Timing: Day 3, variable, depending on the method**

The data received from the Seahorse XF96 Analyzer can be normalized for the seeded cell number (17) or any other parameter of choice, e.g., the protein concentration per well (18).

**Figure 3:**
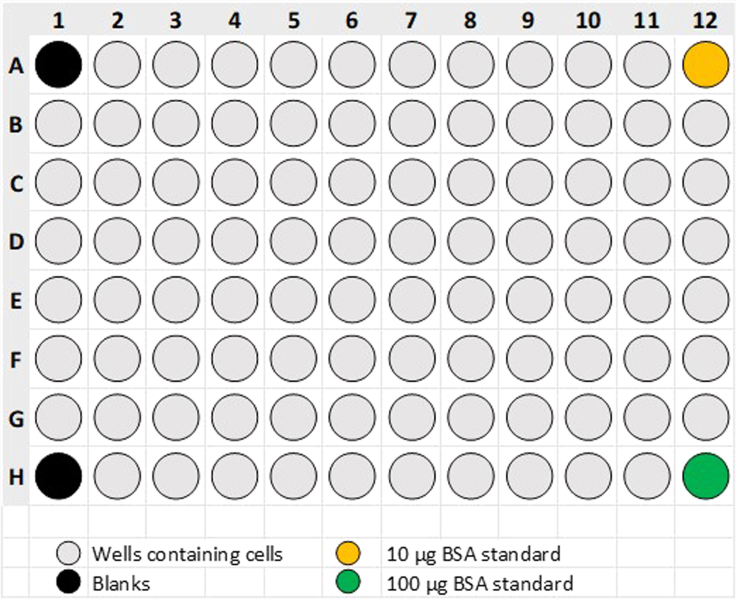
Typical layout used for determination of the protein concentration per well

**Figure 4:**
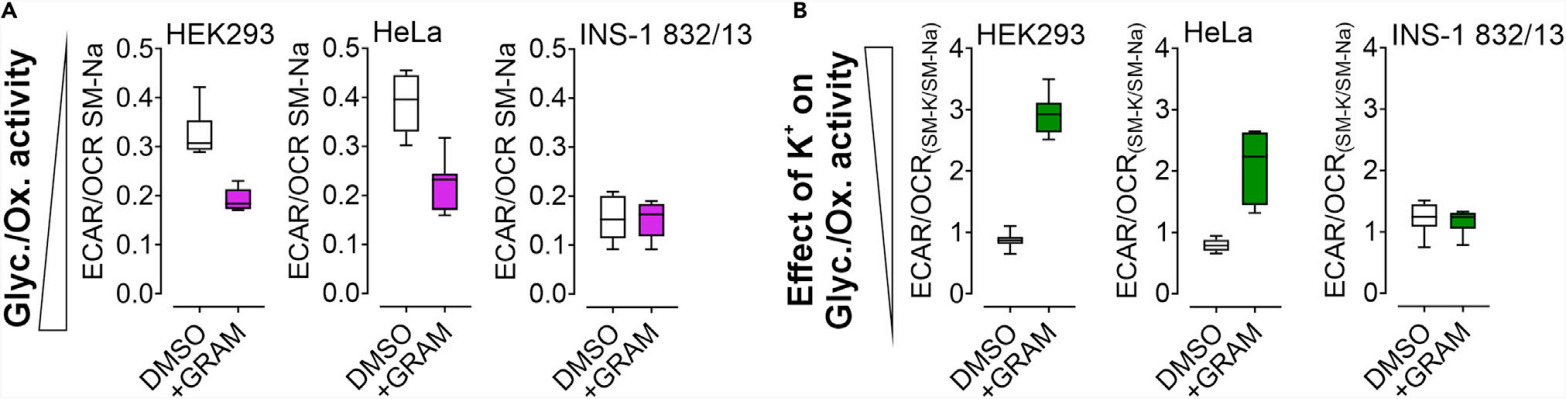
Typical results received from the experiments Calculation of the results as described in this protocol will give you the ECAR/OCR ratio of control cells (white boxes) and gramicidin treated, i.e., K^+^ depleted, cells (magenta boxes), and the effect of high K^+^ on this ECAR/OCR ratio (B). High ratios in (A) indicate high glycolytic vs. oxidative activity, low ratios indicate low glycolytic vs. oxidative activity. Ratios above 1 in (B) indicate a stimulatory, ratios below 1 an inhibitory effect of K^+^ on glycolytic vs. oxidative activity. The presented findings indicate for INS-1 832/13 that their metabolic activity is independent on [K^+^]_i_, while HeLa and HEK293 cells show a dependency on [K^+^]_i_. Boxes indicate the median and the first and third quartile. Lower and upper whiskers indicate 5-95 percentile. Part of the figure has been published in Bischof et al. (2021). Figure reuse with permission from Bischof et al. (2021).
    iii. To analyze for a reconstituting effect of K^+^ on gramicidin treated cells, average the ECAR/OCRs for SM-0K^+^ medium of each cell line treated with DMSO or gramicidin, respectively, and divide each value of SM-140K^+^ medium of the corresponding condition (DMSO/ gramicidin) by the averaged value. Values close to 1 indicate, that high K^+^ does not influence ECAR/OCR ratio, while values lower than 1 indicate an inhibitory, and values higher than 1 a stimulatory effect of K^+^ on ECAR/OCR ratio (Figure 4B). Troubleshooting 6 and 7.

17. The easiest way to normalize the data is to use the Seahorse Wave Desktop software and normalizing the data for the seeded cell number.
  a. Open the data from the Seahorse XF96 Analyzer in the Seahorse Wave Desktop software.
  b. The software will show you the results/ raw data from the run.
  c. Select “normalize” in the top bar within the software.
  d. Enter the seeded cell number / 10^3^ for each well in the table and confirm the normalization by selecting “apply”.
  e. After confirmation, the software directly displays the normalized OCR and ECAR values over-time.

18. Alternatively, in cases where cell detachment after washing was observed, Seahorse data can be normalized for the protein concentration per well to correct for reduced cell numbers.
  a. After finishing the Seahorse assay, take the Seahorse XF96 Cell Culture Microplate and the Seahorse XF96 Sensor Cartridge from the device and discard the Sensor Cartridge.
  b. Carefully remove the supernatants of the cells without disturbing the cells at the bottom. We recommend removing the medium by pipetting.
  c. Air-dry the Seahorse XF96 Cell Culture Plate at room temperature (20°C–25°C) for at least 1 h to remove any residual medium.
  d. After all of the medium has evaporated, freeze the Seahorse XF96 Cell Culture Plate over-night at -80°C.
  e. The next day, take the plate from the -80°C freezer and thaw the plate at room temperature (20°C–25°C) for at least 1 h.
  f. After the plate thawed, put the Seahorse XF96 Cell Culture Plate into the -80°C freezer again for at least 2 h and repeat the freeze-thaw cycle two more times.
  g. After 3 consecutive freeze-thaw cycles, the plate can be used for protein-determination after the 3^rd^ thawing process. A typical layout used for protein concentration determination is displayed in Figure 3.
    i. Prepare the working reagent (WR) of the Pierce BCA Protein Assay Kit by mixing 50 parts of BCA reagent A with 1 part of BCA reagent B. As 30 μL will be used per well, mixing 3 mL of BCA reagent A with 60 μL of BCA reagent B will yield enough WR for 96 protein determinations in the Seahorse XF96 Cell Culture Plate.
    ii. To each well of the Seahorse XF96 Cell Culture Plate except wells A12 and H12, which are used for the generation of the calibration curve, add 50 μL of ddH_2_O to solubilize the cell fragments.
    iii. Put 5 μL of a 2 mg/mL BSA stock solution from the Pierce BCA Protein Assay Kit in well A12 and add 45 μL of ddH_2_O, yielding a protein amount of 10 μg per well.
    iv. Put 50 μL of a 2 mg/mL BSA stock solution from the Pierce BCA Protein Assay Kit in well H12, yielding a protein amount of 100 μg per well.
    v. After all wells are pre-filled with 50 μL of solution, add 30 μL of the WR per well and incubate the plate at 37°C without CO_2_ for 30 min.
    vi. After 30 min of incubation, read absorbance at 562 nm.
  h. Calculate the protein concentration per well. We recommend using Microsoft Excel for calculation.
    i. Subtract the average of wells A1 and H1 (blank wells) from every other well.
    ii. Generate the calibration curve of the BSA standards by plotting the blank corrected absorbance values of wells A12 and H12 over the respective protein concentration (10 μg / 100 μg). Using Microsoft Excel, generate a linear function through these points and show the equation which will be in the following format:
      y=k∗x+d
      Where:
      y= Absorbance value
      k= Slope of the function
      d = Axis section
    iii. Solve the equation for x and calculate the protein concentration for each well by using the absorbance value as x. The final equation for protein concentration calculation will be:
      $$x=\frac{y-d}{k}$$
  i. Having calculated the protein concentration per well, open the data from the Seahorse XF96 Analyzer in the Seahorse Wave Desktop software.
  j. The software will show you the results/ raw data from the run.
  k. Select “normalize” in the top bar within the software.
  l. Enter the calculated protein concentrations in μg for each well in the table and confirm the normalization by selecting “apply”.
  m. After confirmation, the software directly displays the normalized OCR and ECAR values over-time.

19. Analyze the normalized data using a program of choice. We recommend using Microsoft Excel.
  a. Export the data to Microsoft Excel.
  b. The easiest representation is via the “Normalized Rate (Columns)” paragraph.
    i. For analysis of basal ECAR and OCR values of DMSO vs. gramicidin treatment, take the average of several time-points that were measured without injection (e.g., first 3 values) for each well.
    ii. For analysis of basal ECAR/OCR values of DMSO vs. gramicidin treatment, divide the averaged ECARs by the averaged OCRs for each well (Figure 4A).

## Expected outcomes

Performing the experiments and analysis as outlined above will give you the following information:

Do my cells of interest show a gramicidin treatment, i.e., K^+^ sensitive, metabolic activity?

Can the potential K^+^ sensitive metabolic activity be restored by re-addition of a high [K^+^]?

We have recently demonstrated, that affecting the subcellular K^+^ homeostasis, also in a gramicidin free manner, significantly modulates cell metabolic activity, as the activation of a voltage-gated potassium channel, K_V_1.3, in HEK293 cells also modulated the glycolytic activity within these cells (Bischof et al., 2021). As cancers are frequently associated with alterations in their K^+^ channel expression patterns (Huang and Jan, 2014), and the presence of such channels drives cancer malignancy, mostly, however, by unknown means (Felipe et al., 2012; Mohr et al., 2019, Mohr et al., 2020), a K^+^ stimulated metabolic activity might link the alterations in K^+^ channel expression patterns with tumor progression. Whether K^+^ channels can directly drive cancer cell proliferation by stimulating HKII dependent glycolysis remains, however, elusive. Nevertheless, based on recent results of a K^+^ dependent HKII activity, specifically addressing K^+^ channels in cancer cells may affect their metabolic activity and, hence, proliferative activity. Interestingly, besides our report of HKII representing a metabolic, K^+^ dependent enzyme, earlier reports also demonstrated a K^+^ dependency of, for example, the pyruvate kinase (Kachmar and Boyer, 1953). Consequently, whether targeting the intracellular K^+^ homeostasis represents feasible 1) to target cancer cell proliferation and 2) to specifically affect (cancer) cell metabolism by targeting HKII or also via modulating the activity of other enzymes remains elusive but might be aim of future studies.

## Quantification and statistical analysis

Data can be represented and analyzed using any program of choice. We recommend using and plotting every single technical replicate for analysis. Experiments should, however, at least be replicated for a minimum of 3 times on 3 independent days to ensure data consistency and reproducibility.

We recommend testing the data for normal distribution to choose the proper statistical test. Usually, an Unpaired t-test (if data are normally distributed) or a Mann-Whitney-U test (if data are not normally distributed) should be used for pairwise comparison of SM-0K^+^ DMSO vs. SM-0K^+^ gramicidin and SM-140K^+^ DMSO vs. SM-140K^+^ gramicidin.

## Limitations

First, if cells are very resistant to gramicidin treatment, it may be difficult to permeabilize them for K^+^. To ensure the gramicidin sensitivity of cells, it may hence be helpful to conduct intracellular measurements of K^+^ using, for example, recently developed, genetically encoded K^+^ indicators, the GEPIIs (Bischof et al., 2017).

Second, if cells show no adherence and grow in suspension, the protocol may become more difficult and more laborious. In this case, pre-coating of the wells, e.g., with Cell-Tak or Poly-L-Lysine may be helpful.

## Troubleshooting

### Problem 1

The number of cells listed in this protocol does not work as cells are either too confluent (>95%) or show too low density under the microscope (<80%) (step 14).

### Potential solution

Adjust the cell number based on your observations. If cell density seemed to low increase the number of seeded cells and vice versa.

### Problem 2

No ECAR or OCR values are detected by the Seahorse XF96 Analyzer (step 15).

### Potential solution

Be more careful during the cell preparation as cells may have detached during repeated washing. Alternatively, wells may be coated Cell-Tak cell and tissue adhesive to increase cell adherence. Therefore, 2.5 mL of a 22.4 μg/mL of Cell-Tak in PBS solution are prepared and each well of the Seahorse XFe 96-well cell culture plate is incubated with 25 μL of the solution at room temperature (20°C–25°C) for 20 min prior to cell seeding. Please also see https://www.agilent.com/cs/library/technicaloverviews/public/5991-7153EN.pdf

### Problem 3

Seahorse raw data show a large variability from well to well (step 15).

### Potential solution

Make sure that cell suspension is homogeneous when cells are seeded. Additionally, be more careful during the cell preparation as cells in single wells may have detached during repeated washing. Alternatively, wells may be coated Cell-Tak cell and tissue adhesive to increase cell adherence.

### Problem 4

Cells look fine prior to starting the experiment when observing under the microscope. When measurement is started, however, no ECAR or OCR is detected (step 15).

### Potential solution

Cell treatment with gramicidin +/- K^+^ might be too harsh. Try to adjust the gramicidin concentration by gradually lowering. Additionally, try other [K^+^] as 0 or 140.0 mM of K^+^ might be too harsh.

### Problem 5

The baseline during the measurement is not stable but increases or decreases initially or continuously (step 15).

### Potential solution

Baselines that are unstable at the beginning but equilibrate after 2 or 3 measurement cycles frequently indicate bad/ too short equilibration phases of the cells in the seahorse media. If baseline continuously declines, cell detachment/ death might be the reason. Try to shorten gramicidin incubation times under these conditions, in order to keep cell-stress as short as possible.

### Problem 6

Seahorse raw data look fine, but upon normalization for the protein concentration data starts to scatter (step 19).

### Potential solution

Large deviations and scattering data may be caused by problems with the method of normalization, as either the determination of the protein concentration was wrong (if data were normalized for protein concentration) or cells have detached upon washing (if data were normalized for cell number). In both cases, try to normalize for another parameter.

### Problem 7

Results show low reproducibility from day to day (step 19).

### Potential solution

Try to keep as many parameters as possible constant including cell seeding and cultivation conditions, volumes, solvents and incubation times. Make sure that cells are not in a too high passage and do not use cells directly after thawing – cultivate cells for at least 3 passages prior to performing the experiments. Also check all media for potential contamination. Ensure to mix the gramicidin containing seahorse media well, as gramicidin is initially precipitating in aqueous solutions.

## Resource availability

### Lead contact

Further information and requests for resources and reagents should be directed to and will be fulfilled by the lead contact, Roland Malli, roland.malli@medunigraz.at.

### Materials availability

This study did not generate new unique reagents.

## Data Availability

Original/source data for the paper is available from the lead contact upon request.
